# Predictors of survival of therapeutic hypothermia based on analysis of a consecutive American inner-city population over 4 years

**DOI:** 10.1186/cc14508

**Published:** 2015-03-16

**Authors:** BR Roberts, HT Toca, JM Martinez

**Affiliations:** 1 Louisiana State University Health Sciences Center - New Orleans, LA, USA

## Introduction

Therapeutic hypothermia (TH) is the international standard of care for all comatose patients after cardiac arrest, but criticism focuses on poor outcomes. We sought to develop criteria to identify American urban patients more likely to benefit from TH.

## Methods

A retrospective chart review of 107 consecutive adults undergoing TH in downtown New Orleans from 2010 to 2014 yielded records for 99 patients with all 44 survivors or families contacted up to 4 years.

## Results

Sixty-nine males and 38 females with a mean age of 60.2 years showed 63 dead (58%) and 44 survivors (42%). Presenting cardiac rhythm was divided into shockable (pulseless ventricular tachycardia, ventricular fibrillation) and nonshockable (pulseless electrical activity, asystole). Presenting in shockable rhythms with ROSC <20 minutes were 21 patients with 15 (71%) survivors (*P *= 0.001). Time >20 minutes until ROSC in shockable rhythms had five patients with three survivors (78%, *P *= 0.001). Presenting in nonshockable rhythms with ROSC <20 minutes were 54 patients with 18 survivors (33%, *P *= 0.001). ROSC >20 minutes in nonshockable rhythms had 19 patients with two survivors (8%, *P *= 0.001). Survivors of shockable rhythms showed 19 (100%) living post TH. Fifteen survivors (79%, *n *= 19, *P *= 0.001) had CPC score 1 or 2 with four survivors (21%, *n *= 19) having a CPC score of 3. A total of 25 survived nonshockable rhythm. Acute survival of patients with nonshockable rhythm showed 18 expired <72 hours (72%, *n *= 25) with long-term survival of four patients (5%, *n *= 74) and CPC scores of 1 or 2 (*P *= 0.001). Interestingly, patients with time to ROSC <20 minutes exhibiting more than one loss of sustained ROSC showed 100% mortality (*P *= 0.001). Patients presenting with shockable >20 minutes ROSC had overall survival of 70% (*P *= 0.001), but those undergoing >3 cardiac rhythm changes had 100% mortality (*P *= 0.001).

## Conclusion

Patients presenting with shockable rhythms undergoing TH had overall acute survival of 70% followed by long-term survival of 100% after 4 years. In contrast, patients presenting with nonshockable rhythm had long-term survival of 5%. TH is not recommended.

